# Epidemiology of birth defects in a national hospital-based birth defect surveillance spot in Southern Jiangsu, China, 2014–2018

**DOI:** 10.3389/fmed.2023.1138946

**Published:** 2023-09-12

**Authors:** Ying Zhou, Di Yang, Xueqin Mao, Hua Zhou, Li Wang

**Affiliations:** ^1^Department of Health Emergency, Changzhou Center for Disease Control and Prevention, Changzhou, China; ^2^Department of Obstetrics and Gynecology, Changzhou Maternity and Child Health Care Hospital, Changzhou Medical Center, Nanjing Medical University, Nanjing, China

**Keywords:** birth defects, epidemiology, hospital-based, surveillance, risk factors

## Abstract

**Objective:**

As the only hospital-based national surveillance spot of birth defects (BDs) in Changzhou city located in the economically developed eastern part of China, Changzhou Maternal and Child Health Care Hospital has encountered serious challenges in BD prevention. This study aimed to describe the epidemiology of total BDs born in the hospital from 2014 to 2018.

**Methods:**

The data were collected from the national hospital-based birth defect surveillance system. BD prevalence was calculated by Poisson distribution. Trends of prevalence and the associations regarding information with BDs were analyzed by Poisson regression.

**Results:**

The reported prevalence of total BDs was 313.92 (95% confidence interval [CI]: 299.59–328.76) per 10,000 perinatal infants (PIs), while the perinatal prevalence of BD was 160.19 (95% CI: 150.00–170.89) per 10,000 PIs. A remarkable uptrend in the prevalence of BDs was noticed with a prevalence rate ratio (PRR) of 1.09 (95% CI: 1.04–1.14) and 1.13 (95% CI: 1.09–1.16), respectively. Congenital heart disease (CHD), cleft lip with or without cleft palate (CL/P), congenital malformation of the kidney (CMK), polydactyly, Down syndrome (DS), cystic hygroma, neural tube defect (NTD), and congenital talipes equinovarus (CTE) were common types of total BDs. Mothers living in the urban area (PRR = 1.67, 95% CI:1.50–1.87), male fetuses (PRR = 1.16, 95% CI: 1.05–1.28), and maternal age younger than 20 (PRR = 2.28, 95% CI: 1.60–3.25) and 25 years (PRR = 1.41, 95% CI: 1.22–1.63) or older than 35 years (PRR = 1.18, 95% CI: 1.00–1.40) were risk factors for BD occurrence.

**Conclusion:**

The reported prevalence of total BDs was nearly two times higher than the perinatal prevalence of BDs in PIs, and the ranks of total BDs and BDs in PIs were different. Mothers living in the urban area, male fetuses, and maternal ages younger than 25 or older than 35 years were risk factors for BD incidence. Thus, improving prenatal examination technology, expanding the surveillance time quantum of BDs, and keeping maternal health may be warranted.

## 1. Introduction

Birth defects (BDs), also known as congenital anomalies, are structural, functional, or metabolic anomalies that occur during intrauterine life and can be identified prenatally, at birth, or sometimes later in infancy (1). BDs have caused high morbidity and mortality of fetuses and infants and significant economic burden to both families and society and have been a global public health issue (2). The World Health Organization (WHO) has reported the prevalence of BDs at 47.2, 55.7, and 64.2 per 1,000 live births in developed, middle-income, and low-income countries, respectively (3). The estimated prevalence of BDs in China has been ~40–60 per 1000 live births, which is close to the level of middle-income countries (4). Meanwhile, the prevalence and rank of BDs have undergone tremendous changes. In the recent century, a significant increase in BD prevalence has been noticed in China, Korea, and Uganda (5–7). The prevalence of several BD subgroups, such as congenital heart disease (CHD), increased significantly, while the prevalence of neural tube defect (NTD) substantially decreased. Changes in prevalence caused rank alteration of BDs (6). Effective detection and full understanding of BDs are useful ways to prevent them, and describing the epidemiology of BDs can aid in implementing and evaluating preventive interventions (8).

There are two types of national surveillance systems in China (9). One is the hospital-based surveillance system, which is used to track the total number of BDs, including those in born children or terminated pregnancies. However, for higher requirements of medical level and longer monitoring time quantum of surveillance hospitals, most hospitals in China are still monitoring BDs in perinatal infants (PIs are infants aged between 28 weeks of gestation and 7 days after birth). Thus far, many studies have described the epidemiology of BDs in PIs of China (5, 10), but few had information regarding total BDs, which could underestimate the prevalence of BDs, especially regarding certain major prenatally diagnosed malformations (10). Therefore, it is urgent to evaluate total BDs.

Changzhou Maternal and Child Health Care Hospital is the only specialized hospital of obstetrics in Changzhou city, where >10,000 neonates are delivered every year. Meanwhile, as the only national hospital-based birth defect surveillance spot in Changzhou, the hospital reports total BDs of the whole gestational period in the national surveillance system. This study aimed at investigating and comparing the epidemiology of BDs in PIs and total BDs by using data from the national hospital-based birth defect surveillance system from 2014 to 2018.

## 2. Materials and methods

### 2.1. Study population

All pregnant women who delivered in Changzhou Maternal and Child Health Care Hospital between 2014 and 2018 were monitored. Live births within 7 days, stillbirth, and termination of pregnancy (ToP) at any gestational age following the perinatal diagnosis of BDs were recorded. Pregnancy records were anonymized. Participants' consent forms were achieved when doctors filled in the “Birth Defects Registration Form” after their delivery. The study complied with the World Medical Association Declaration of Helsinki and was approved by the Ethics Committee of Changzhou Maternity and Child Health Care Hospital.

### 2.2. Criteria of BD diagnosis

The criteria for BD diagnosis were based on the “Maternal and Child Health Monitoring Manual in China”. Anomalies were diagnosed by physical and auxiliary examinations, such as prenatal ultrasonography, and by professional obstetric or neonatal doctors of Changzhou Maternal and Child Health Care Hospital. Complex anomalies were diagnosed through expert consultation. In the surveillance system, 24 types of BDs are registered in detail, and other types of BDs are classified as “others” (10). Only types of BDs that ranked the first 16th were shown in this study, while the other types of BDs were rare. In addition, cleft lip with cleft palate and cleft lip without cleft palate were merged as cleft lip with or without cleft palate (CL/P). Congenital malformation of the kidney (CMK), cystic hygroma, single umbilical artery, congenital atresia of the intestine, subcutaneous edema, visceral inversion, holoprosencephaly, spine arrangement disorder, congenital club hands, Klinefelter's syndrome, and gastrointestinal obstruction were separated from others. BDs were coded according to the International Classification of Disease and Related Health Problems, 10th Revision (ICD-10) (9).

### 2.3. Data collection and quality control

The National Health and Family Planning Commission has formulated the surveillance data, including the “Birth Defects Registration Form” of livebirth, stillbirth, and ToP of the whole gestational period with BDs and a quarterly table with information on PIs. These data were collected and completed by experienced obstetricians and pediatricians of the hospital. Cases diagnosed by auxiliary examination needed to be confirmed by clinicians after delivery or elective termination. Every case form noted the maternal and neonatal information and BD diagnosis, among other data. Each quarterly table included the number of PIs in each maternal age group, the number of stillbirths, neonatal deaths, and BDs in PIs in each quarter. The case form and the quarterly table were reported both on paper and online. The staff of the Tianning Maternal and Child Health and Family Planning Service Center received and input all the information to the surveillance system monthly and audited them quarterly. The staff of the Changzhou Maternal and Child Health Care Hospital received and audited the information quarterly. Quality controls of the data were examined once every quarter at the district level, half-yearly at the city level, and yearly at the province level to ensure completeness and accuracy of the data and reduction of underreported errors.

### 2.4. Statistical analysis

The prevalence and 95% confidence interval (CI) of BDs were calculated by Poisson distribution. In prevalence calculation, the denominator remained the same: the number of PIs. For perinatal prevalence, the numerator was the number of BDs in PIs, and for reported prevalence, the numerator was the number of total BDs regardless of gestational age. The prevalence of 16 leading BDs was also calculated and ranked in descending order. Univariate Poisson regression was performed to identify the changing trends of BD prevalence by year, and multivariable Poisson regression was used to detect associations between regarding characteristics and BDs. R version 4.1.3 (the Comprehensive R Archive Network: http://cran.r-project.org) was used for the data analysis. A P-value of <0.05 was considered statistically significant.

## 3. Results

As shown in Table 1, during the study period, 57,371 PIs were registered, and 919 BDs in PIs were diagnosed, resulting in a perinatal prevalence of 160.19 (95% CI: 150.00–170.89) per 10,000 PIs. Univariate Poisson regression showed that the perinatal prevalence of BDs increased significantly during the 5-year study period (prevalence rate ratio [PRR] = 1.09, 95% CI: 1.04–1.14). The perinatal prevalence of BDs in the urban area was significantly higher than that in the rural area (183.55 *vs*. 115.97 per 10,000 PIs, PRR = 1.58, 95% CI: 1.36–1.84). The perinatal prevalence of BDs in male fetuses was significantly higher than that in female fetuses (176.44 *vs*. 140.66 per 10000 PIs, PRR = 1.25, 95% CI: 1.10–1.43). The perinatal prevalence of BDs in mothers aged 30–34 years was the lowest in this study; thus, it was set as a reference. The perinatal prevalence of BDs in mothers aged <20 years 20–24 years was significantly higher than that in the reference group (314.34 vs. 149.45 per 10,000 PIs, PRR = 2.10, 95% CI: 1.27–3.50 and 192.12 *vs*. 149.45 per 10000 PIs, PRR = 1.29, 95% CI: 1.05–1.57, respectively). However, a significant difference between the perinatal prevalence of BDs in mothers aged >35 years and that in the reference group was not identified. In addition, the perinatal prevalence of BDs in multiple births was significantly higher than that in singletons (243.19 *vs*. 155.47 per 10000 PIs, PRR = 1.56, 95% CI: 1.24–1.98).

**Table 1:** Perinatal prevalence of BDs according to different characteristics.

**Characteristics**		**BDs of PIs (n)**	**PIs (n)**	**Prevalence of BDs per 10000 PIs (95%CI)**	**PRR (95%CI)**
Year	2014	161	12,197	132.00 (112.40, 154.04)	1.09 (1.04, 1.14)
	2015	163	10,742	151.74 (129.34, 176.91)	
	2016	214	13,256	161.44 (140.53, 184.58)	
	2017	194	11,134	174.24 (150.58, 200.56)	
	2018	187	10,042	186.22 (160.48, 214.91)	
	Total	919	57,371	160.19 (150.00, 170.89)	/
Region	Urban	689	37,538	183.55 (170.10, 197.78)	1.58 (1.36, 1.84)
	Rural	230	19,833	115.97 (101.46, 131.96)	Reference
Sex	Male	533	30,208	176.44 (161.78, 192.08)	1.25 (1.10, 1.43)
	Female	382	27,158	140.66 (126.91, 155.50)	Reference
Maternal age	<20	16	509	314.34 (179.67, 510.47)	2.10 (1.27, 3.50)
	20~	173	9005	192.12 (164.55, 222.97)	1.29 (1.05, 1.57)
	25~	418	27,284	153.20 (138.87, 168.62)	1.03 (0.87, 1.21)
	30~	212	14,185	149.45 (130.01, 170.98)	Reference
	35~	100	6,388	156.54 (127.37, 190.40)	1.05 (0.83, 1.33)
Type of pregnancy	Multiple	75	3,084	243.19 (191.28, 304.84)	1.56 (1.24, 1.98)
	Singleton	844	54,287	155.47 (145.16, 166.32)	Reference

After combining the data on BDs at <28 weeks of gestation, the reported prevalence of total BDs analyzed from years and potential risk factors is summarized in Table 2. The reported prevalence of total BDs was 313.92 (95% CI: 299.59–328.76) per 10,000 PIs, nearly two times higher than perinatal prevalence. The reported prevalence of total BDs increased remarkably during the study period (PRR = 1.13, 95% CI: 1.09–1.16). In addition, the reported prevalence of total BDs was significantly higher in the urban *versus* rural areas (364.70 vs. 217.82 per 10000 PIs, PRR = 1.67, 95% CI: 1.50–1.87) and in male fetuses *versus* female fetuses (307.87 vs. 265.85 per 10000 PIs, PRR = 1.16, 95% CI: 1.05–1.28). Furthermore, with reported prevalence in mothers aged 30–34 years set as the reference group, PRRs were 2.28 (95% CI: 1.60–3.25) in mothers aged <20 years and 1.41 (95% CI: 1.22–1.63) in mothers aged 20–24 years. Moreover, the difference between the reported prevalence of BDs in mothers aged > 35 years and the reference group was moderate (PRR = 1.18, 95% CI: 1.00–1.40). However, a significant difference between multiple births and singletons was not identified.

**Table 2:** Reported prevalence of total BDs according to different characteristics.

**Characteristics**		**Total BDs (n)**	**PIs (n)**	**Reported prevalence of BDs (95%CI)**	**PRR (95%CI)**
Year	2014	273	12,197	223.83 (198.06, 252.01)	1.13 (1.09, 1.16)
	2015	340	10,742	316.51 (283.76, 352.01)	
	2016	408	13,256	307.79 (278.64, 339.15)	
	2017	389	11,134	349.38 (315.52, 385.89)	
	2018	391	10,042	389.36 (351.72, 429.94)	
	Total	1801	57,371	313.92 (299.59, 328.76)	/
Region	Urban	1369	37,538	364.70 (345.63, 384.54)	1.67 (1.50, 1.87)
	Rural	432	19,833	217.82 (197.76, 239.36)	Reference
Sex	Male	930	30,208	307.87 (288.39, 328.31)	1.16 (1.05, 1.28)
	Female	722	27,158	265.85 (246.81, 285.97)	Reference
Maternal age	<20	33	509	648.33 (446.28, 910.50)	2.28 (1.60, 3.25)
	20~	361	9005	400.89 (360.60, 444.45)	1.41 (1.22, 1.63)
	25~	789	27,284	289.18 (269.35, 310.08)	1.02 (0.90, 1.15)
	30~	403	14,185	284.10 (257.04, 313.24)	Reference
	35~	215	6,388	336.57 (293.08, 384.69)	1.18 (1.00, 1.40)
Type of pregnancy	Multiple	93	3,084	301.56 (243.40, 369.43)	0.96 (0.78, 1.18)
	Singleton	1708	54,287	314.62 (299.88, 329.91)	Reference

Table 3 shows the rank, prevalence, and proportion of different types of BDs. During the study period, 10 major subtypes of total BDs were CHD, CL/P, CMK, polydactyly, Down syndrome (DS), cystic hygroma, NTD/congenital talipes equinovarus (CTE), hypospadias, congenital hydrocephalus, and syndactyly. CHD, CL/P, CMK, DS, cystic hygroma, NTD, CTE, congenital hydrocephalus, gastroschisis, and omphalocele/limb reduction defects (LRDs) were the 10 most common subtypes of BDs among fetuses aged <28 weeks of gestation. However, among the PIs, the 10 most common subtypes of BDs were CHD, polydactyly, CMK, hypospadias, syndactyly, CL/P, other malformation of external ear (OMEE), congenital hydrocephalus/congenital atresia of the rectum and anus, cleft palate without a cleft lip, and CTE, which were different from those in total BDs.

**Table 3:** Major 16 subtypes of BDs.

**Rank**	**Total BDs**				**BDs in PIs**				**BDs**<**28 gestational weeks**		
	**BDs**	**Count**	**Prevalence (95%CI)**	**Proportion (%)**	**BDs**	**Count**	**Prevalence (95%CI)**	**Proportion (%)**	**BDs**	**Count**	**Proportion (%)**
1	Congenital heart defects	486	84.71 (77.35, 92.59)	26.99	Congenital heart defects	238	41.48 (36.38, 47.10)	25.90	Congenital heart defects	248	28.12
2	Cleft lip with or without cleft palate	179	31.20 (26.80, 36.12)	9.94	Polydactyly	143	24.93 (21.01, 29.36)	15.56	Cleft lip with or without cleft palate	139	15.65
3	Congenital malformation of the kidney	177	30.85 (26.47, 35.75)	9.83	Congenital malformation of the kidney	91	15.86 (12.77, 19.7)	9.90	Congenital malformation of the kidney	86	9.75
4	Polydactyly	149	25.97 (21.97, 30.49)	8.27	Hypospadias	63	10.98 (8.44, 14.05)	6.86	Down syndrome	81	9.18
5	Down syndrome	93	16.21 (13.08, 19.86)	5.16	Syndactyly	49	8.54 (6.32, 11.29)	5.33	Cystic hygroma	80	9.07
6	Cystic hygroma	82	14.29 (11.37, 17.74)	4.55	Cleft lip with or without cleft palate	40	6.97 (4.98, 9.49)	4.35	Neural Tube Defects	63	7.26
7	Neural Tube Defects/Congenital talipes equinovarus	71	12.38 (9.67, 15.61)	3.94	Other malformation of external ear	38	6.62 (4.69, 9.09)	4.13	Congenital talipes equinovarus	44	4.99
8	Hypospadias	63	10.98 (8.44, 14.05)	3.50	Congenital hydrocephalus /Congenital atresia of the rectum and anus	31	5.40 (3.67, 7.67)	3.37	Congenital hydrocephalus	29	3.29
9	Congenital hydrocephalus	60	10.46 (7.98, 13.46)	3.33	Cleft palate without cleft lip	28	4.88 (3.24, 7.05)	3.05	Gastroschisis	25	2.83
10	Syndactyly	54	9.41 (7.07, 12.28)	3.00	Congenital talipes equinovarus	27	4.71 (3.10, 6.85)	2.94	Omphalocele / Limb reduction defects	23	2.61
11	Other malformation of external ear	38	6.62 (4.69, 9.09)	2.11	Microtia	23	4.01 (2.54, 6.02)	2.50	Single umbilical artery	16	1.81
12	Limb reduction defects/Congenital atresia of the rectum and anus	35	6.10 (4.25, 8.48)	1.94	Congenital esophageal atresia	13	2.27 (1.21, 3.87)	1.41	Subcutaneous edema	15	1.70
13	Cleft palate without cleft lip	29	5.05 (3.39, 7.26)	1.61	Limb reduction defects	12	2.09 (1.08, 3.65)	1.31	Visceral inversion	13	1.47
14	Gastroschisis	28	4.88 (3.24, 7.05)	1.55	Single umbilical artery	11	1.92 (0.96, 3.43)	1.20	Holoprosencephaly	12	1.36
15	Single umbilical artery	27	4.70 (3.10, 6.85)	1.50	Congenital atresia of intestine	10	1.74 (0.84, 3.21)	1.09	Spine arrangement disorder / Congenital club hands	10	1.13
16	Omphalocele	24	4.18 (2.68, 6.22)	1.33	Gastrointestinal obstruction	9	1.57 (0.72, 2.98)	0.98	Klinefelter' s syndrome	9	1.02

## 4. Discussion

In the current study, the perinatal prevalence of BDs was 160.19 per 10,000 PIs, which is higher than the average level in Changzhou city (71.509, 2014–2018) (5) and Longgang district of Shenzhen (134.3, 2003–2009) (11) but lower than that in Hunan province (191.84, 2005–2014) (10). The reported prevalence of total BDs was 313.92 per 10,000 PIs, which is higher than that in Norway (290, 1980–2012) (12), and the average level of Jiangsu province (135.53, 2010–2014) (13) but lower than that in Korea (446.3, 2008–2014) (6). The discrepancy mentioned above might be caused by differences in regions, monitoring time quantum, and types of BDs included in the studies (6). Furthermore, treating more complicated pregnant women and having fewer underreported errors might be the reason for this higher prevalence than that of the average level of Changzhou city (5) and Jiangsu province (13). In addition, the apparent upward trend in the prevalence of BDs was detected during the study period, which is the same as that in Korea (6) and Uganda (7), which might be caused by environmental pollution, diagnostic technique improvement, and fewer underreported errors (5, 7). Therefore, avoiding exposure to environmental pollutants and improving diagnosis skills are critical to prevent, detect, and treat BDs on time (14).

As shown in this study, the reported prevalence of total BDs (313.92) was nearly two times higher than the perinatal prevalence of BDs in PIs (160.19), meaning it underestimates the total BDs by 50% if only BDs in PIs were monitored. This limitation has been reported by a Japanese study (15). Meanwhile, sustained surveillance after birth also detected some neonatal diseases, such as congenital hypothyroidism, which probably could not be found among PIs (9). Meanwhile, as shown in Table 3, except for polydactyly, the first seven types of BDs were the same between BDs at <28 weeks of gestation and total BDs but different from those in PIs, which proved that the reported disease spectrum of BDs was erroneous if only BDs in PIs were monitored. In addition, most BDs in PIs were less harmful or treatable. As researchers from the United States have noted, advancing diagnosis skills allow for BD detection before 28 weeks of gestation, and most pregnant women with severe BDs might choose the ToP (16). This attitude to BDs has also been found among women in Hong Kong, China (17). Additionally, in this study, it was estimated that nearly half of CHD, more than half of CL/P and CTE, and almost all lethal and residual BDs, such as DS and cystic hygroma, resulted in ToP before 28 weeks of gestation, according to the number of total BDs and BDs in PIs. Thus, expanding the time quantum of BD surveillance from conception is essential to estimate the exact epidemiology of BDs to develop better prevention measures. Moreover, it is necessary to enhance the technology of prenatal diagnosis to reduce the number of infants born with severe BDs and carefully plan medical care before and after birth, to improve survival rates and quality of life of treatable cases (18).

Similar to the findings of several studies all over the world, CHD ranked first among total BDs (6, 10, 19). A systematic review including 260 studies has revealed that the reported prevalence of CHD globally continues to increase, and the prevalence of CHD in Asia is higher than that in Europe and America (20). This increase is attributed to the advance and accessibility of detection methods such as perinatal B-mode ultrasonography (21). Potential risk factors also include air pollution and toxic chemicals (22). CL/P ranked second among total BDs in this study. The global average prevalence of CL/P has been approximately 0.794‰, with geographical and ethnic variation (23). CL/P imposes serious physical and mental problems on pediatric patients and a huge financial burden on their families and society (24). The etiology of CL/P may involve genetic factors, environmental factors, such as maternal smoking, alcohol consumption, or exposure to pesticides during the first trimester, and gene–environment interactions (25). The third highest type of BDs in this study was CMK. Some studies have suggested that the high incidence of CMK might be attributed to complex interactions between genetic and environmental factors, such as maternal obesity or diabetes, maternal drug intake, and chlorination disinfection byproduct in drinking water (6, 26). Polydactyly was also a common type of BD in this study, and it was the most common BD in PIs of Changzhou city (5). Gestational hypertension, maternal infectious disease, paternal smoking, and genetic factors have been associated with the incidence of polydactyly (27). The fifth highest type of BDs was DS in this study. DS is a genetic disorder of the chromosomes, with an estimated birth prevalence of 14 per 10,000 live births (28). It leads to early miscarriage, fetal death, learning disabilities, and other health concerns (29). Some studies have shown an uptrend in the incidence and termination among fetuses with DS. Meanwhile, researchers have indicated that advanced maternal age, male fetuses, and developed area were associated with the incidence of DS (30). Cystic hygroma ranked sixth among all types of BDs in this study. Cystic hygroma is a malformation of the lymphatic system with an incidence of 12.5 per 10,000 pregnancies. In total, 50% of cystic hygroma is associated with chromosomal abnormalities, mainly Turner syndrome, and 40% of cystic hygroma occurs in genetic syndromes, meaning that 90% of cases face fetal death, while the remaining 10% of cases probably have a good prognosis (31). NTD and CTE ranked seventh in this study. NTD is a structural disorder of the central nervous system and is a major cause of perinatal mortality, child morbidity, and disabilities. NTD is primarily a folate deficiency disease (32). Its incidence in China is 10 times higher than that in the United States and Europe (33). CTE affects 1–3 in 1,000 live births and occurs twice as often in male fetuses (34). Family history, twin births, and maternal alcohol consumption have been reported as risk factors for CTE (35). In addition, similar to other research in China, the reported prevalence of LRD was also high in this research (36). Thalidomide and pre-gestational diabetes are risk factors for LRD while taking folate before and/or during pregnancy is associated with a lower risk of offspring LRD (37). Thus, improvement of prenatal examination, avoidance of exposure to environmental pollutants and pathogenic microorganisms, normal maternal blood pressure and blood glucose maintenance, fertility at an appropriate age, maternal drug application under the guidance of doctors, folate/multivitamin supplements before and during pregnancy, and balanced medical resources are effective measures to prevent and control BDs on time.

It was found that urban area and male fetuses were risk factors for BDs, which is consistent with a previous study (10). The prevalence of higher BDs among male fetuses might be explained by the higher susceptibility of the Y chromosome than the X chromosome and more detectable external genital deformities in male fetuses (38, 39). Stronger overall health awareness, increased accessibility to prenatal examination, and more serious environmental pollution in urban areas might cause higher BD prevalence (6, 10). In addition, compared with the reported prevalence of BDs in mothers aged 30–34 years, the reported prevalence of BDs in mothers aged <20 or 20–24 years was higher in this study. As reported, young maternal age (<20 years) means an increase in accidental pregnancy and ToP, which is a risk factor for BD (40). Meanwhile, maternal age of <25 years is associated with an elevated risk of polydactyly (41). Different from the result in PIs, a moderate association between advanced maternal age (> 35 years) and the reported prevalence of total BDs was detected, which is similar to another study in China (10). On the one hand, advanced maternal age (35–40 years) has been associated with BDs such as CHD and CL/P (42). On the other hand, advanced maternal age indicated a higher possibility of serious BDs, such as chromosome aberration (43). Severe BDs in pregnant women aged > 35 years are generally ToP, and this might illustrate why the association between advanced maternal age and BD incidence in PIs could not be detected to some extent. Although multiple births were demonstrated as a risk factor for BDs in PIs, a significant association between multiple births and total BDs was not detected. A retrospective cohort study reported that there was no difference in the risk of BDs between multiple births and singleton (44). Therefore, the association between multiple births and the risk of BDs warrants further study.

There were several limitations in this study. First, this was a single-hospital study. However, more than one-third of pregnant women in Changzhou city gave birth at Changzhou Maternal and Child Health Care Hospital every year; hence, the data were representative. Second, different types of BDs have different etiology and pathogenesis; therefore, it is better to collect background information on demographic and social economic status and explore risk factors for certain types of BDs. Third, the denominator of the reported prevalence of BDs only included PIs. As the numbers of births before 28 weeks of gestation are smaller compared with those of births after 28 weeks of gestation, the impact on the reported prevalence is expected to be minimal (32). Fourth, early BDs could not document extremely for miscarriage, which has been extremely rare for these studies.

## 5. Conclusion

In summary, a remarkable uptrend in the prevalence of BDs was noticed from 2014 to 2018. The reported prevalence of total BDs was nearly two times higher than the perinatal prevalence of BDs in PIs. CHD, CL/P, CMK, polydactyly, DS, cystic hygroma, NTD, and CTE were normal types of BDs of the whole gestational period. Urban area, male fetuses, and maternal age <25 or >35 years were risk factors for BD occurrence. Improving prenatal examination technology, expanding the surveillance time quantum of BDs in the surveillance system, avoiding exposure to environmental pollutants and pathogenic microorganisms, maintaining maternal health, fertility at an appropriate age, nutrition supplements, and balanced medical resources are effective measures to timely prevent and control BDs. Meanwhile, multi-center research including background information is also needed in future studies.

## Data availability statement

The datasets used and/or analyzed during the current study are available from the corresponding author on reasonable request. Requests to access these datasets should be directed to YZ, 1341628074@qq.com.

## Ethics statement

The studies involving human participants were reviewed and approved by the Ethics Committee of Changzhou Maternal and Child Health Care Hospital. Written informed consent to participate in this study was provided by the participants' legal guardian/next of kin.

## Author contributions

YZ designed the study, conducted statistical analysis, conceptualized, and wrote the initial draft of the manuscript. DY revised the manuscript and investigation. XM and HZ inputted and audited the data. LW summarized the data and wrote the initial draft of the manuscript. All authors read and approved the final manuscript.
